# Analysis of the evolving factors of social media users’ emotions and behaviors: a longitudinal study from China’s COVID-19 opening policy period

**DOI:** 10.1186/s12889-023-17160-y

**Published:** 2023-11-13

**Authors:** Qiaohe Zhang, Jinhua Yang, Tianyue Niu, Kuo-Hsun Wen, Xinhui Hong, YuChen Wu, Min Wang

**Affiliations:** 1 Academy of Fine Arts, Huaibei Normal University, Huaibei, 235000 China; 2 College of Humanities, Tongji University, Shanghai, 200000 China; 3 Academy of Arts & Design, Tsinghua University, Beijing, 10003 China; 4 School of Design, Fujian University of Technology, Fuzhou, 350118 China; 5 Xiamen Academy of Arts and Design, Fuzhou University, Xiamen, 361021 China; 6 College of Humanities and Arts, Macau University of Science and Technology, Macau, 999078 China; 7 School of Design, Jiangnan University, Wuxi, 214122 China

**Keywords:** Emotional evolution, Social media user behavior, Sudden policy changes

## Abstract

The outbreak of the COVID-19 pandemic has triggered citizen panic and social crises worldwide. The Chinese government was the first to implement strict prevention and control policies. However, in December 2022, the Chinese government suddenly changed its prevention and control policies and completely opened up. This led to a large-scale infection of the epidemic in a short period of time, which will cause unknown social impacts. This study collected 500+ epidemic-related hotspots and 200,000+ data from November 1, 2022, to March 1, 2023. Using a sentiment classification method based on pre-trained neural network models, we conducted inductive analysis and a summary of high-frequency words of various emotions. This study focuses on the inflection point of the emotional evolution of social media users and the evolution of “hot topic searches” events and emotional behavioral factors after the sudden open policy. Our research results show that, first of all, the positive emotions of social media users are divided into 4 inflection points and 5 time periods, and the negative emotions are divided into 3 inflection points and 4 time periods. Behavioral factors are different at each stage of each emotion. And the evolution patterns of positive emotions and negative emotions are also different. Secondly, the evolution of behavioral elements deserves more attention. Continue to pay attention: The treatment of diseases, the recovery of personal health, the promotion of festive atmosphere, and the reduction of publicity on the harm of “new crown sequelae and second infections” are the behavioral concerns that affect users’ emotional changes. Finally, it is necessary to change the “hot topic searches” event by guiding the user’s behavioral focus to control the inflection point of the user’s emotion. This study helps governments and institutions understand the dynamic impact of epidemic policy changes on social media users, thereby promoting policy formulation and better coping with social crises.

## Introduction

Since December 2019, the COVID-19 pandemic has had a serious impact on Chinese society and caused a sharp decline in the Chinese economy. To mitigate the spread of the virus, policies of home confinement were implemented. Efforts to limit the spread of the pandemic have led to economic contraction on a global scale [1]. The COVID-19 pandemic is an important source of risk for government governance [2]. Therefore, further research is needed on the measures taken by governments to address the risks posed by the pandemic. This study pays special attention to China’s epidemic opening policy: first of all, it is due to the dual consideration of the huge pressure of domestic epidemic prevention and control and the successful experience of foreign countries. In early December 2023, local governments were still implementing strict epidemic prevention and control policies, but by December 7, the central government announced the “10 New Measures”^1^ opening policy. The announcement of the lockdown lifting policy was very sudden and had the characteristics of suddenness. Secondly, China was the first country to strictly control the spread of the COVID-19 epidemic, and it was also the country that announced the release of the epidemic very late, and implemented an extremely strict epidemic containment policy for three years. It has special research value. Third, since March 2020, under the implementation of strict epidemic prevention, China has not had large-scale epidemic infections, and people’s lives have generally returned to normal. However, the sudden promulgation of the epidemic opening policy made the government and people unable to respond to the large-scale infection in a short period of time, leading to widespread social panic. Therefore, through continuous research for four months before and after the policy is promulgated, analyzing the sudden shift in China’s prevention and control policy will help to study the long-term impact of the new crown policy on China, and can provide useful insights for handling similar public emergencies in the future.

The Chinese government quickly took measures to prevent the spread of COVID-19 [3]. Large-scale lockdowns helped to contain the outbreak. The impact of the COVID-19 pandemic on internet-related industries was relatively small. Under the government’s lockdown policy, residents were unable to have effective offline communication and were forced to be isolated at home [4]. Recent studies have shown that social media users have a significant relationship with epidemic prevention and control. Social media platforms such as Weibo, which have real-time, interactive, and diversified features, have become important media for disseminating hot events, reflecting public opinion, and measuring online public sentiment in China. Therefore, the emotions of internet users are an important aspect of COVID-19-related research [5].

Against the backdrop of the government’s large-scale lockdown policy, the health crisis of COVID-19 has had a negative impact on Chinese citizens. In the first quarter of 2020, China’s national economic output shrank by 6.8%, marking the worst economic performance since China began implementing the national economic accounting system in 1992. The most severely affected industries were accommodation and catering, which contracted by 35.3%, followed by wholesale and retail [6]. This economic downturn directly affected the lives and incomes of residents, causing them to feel anxious and panicked. People longed to return to normal life. Faced with the spread of the epidemic, people also went out to work and engage in normal activities. This is also an emotional challenge and a reality that citizens face. The emotions of citizens in the face of the epidemic directly affect the implementation of government epidemic policies and the recovery and development of the country’s economy. Currently, many governments in various countries have incorporated citizens’ happiness into government decision-making [7], and users’ emotional indicators are a core component of happiness. Users’ emotional health affects the formulation and implementation of government policies [8, 9], and during the COVID-19 pandemic, it is even more important to pay attention to changes and developments in emotions. Numerous researchers have conducted extensive research on the potential impact of public emotions on government lockdown policies during the epidemic [10–12]. Currently, emotional research on epidemic management policies mainly focuses on two aspects:

1. Research on lockdown policies. Natural language processing (NLP) is used to determine the emotional value of online texts and analyze the emotions of Internet users under the blocking policy. The emotional analysis of Internet users shows that depending on the time of the blockade, the mood of Internet users during the blockade period is positive or negative, which is mainly determined by the specific blockade measures. The majority of users support the implementation of lockdown measures and express positive sentiments [5, 13]. As the epidemic develops, the proportion of negative emotions increases. This type of research mainly focuses on the judgment of users’ emotional expression, whether positive or negative emotions, without in-depth analysis of emotional factors [14].
2. Research on opening policy. Such studies show that large numbers of citizens express support for reopening lockdown policies but express emotional concerns about their implementation [15]. This is a contradictory emotional expression that requires specific analysis of the factors that generate user emotions [16]. There are studies analyzing the characteristics and influencing factors of Twitter users’ emotions towards the reopening of the economy [17]. By analyzing Twitter users, as the COVID-19 crisis gradually weakens and control measures against the epidemic are gradually relaxed around the world, users have a positive attitude towards this policy.These studies only analyze the emotional expression of epidemic policies at a macro level and do not discuss the specific elements of emotion in depth. Our study is the first to report on the emotional behavior of social media users during the widespread COVID-19 infection within one month of China’s “China Covid-19 Opening Policy”^2^ (December 1, 2022 to December 20, 2022) [18]. It also discussed the positive attitude at a macro level, including “happy”, “encouragement” and “support”; the negative attitude including: “sadness”, “angry” and “criticism”. This study highlights the affective attitudinal impact of the “China Covid-19 Opening Policy” on social media users. Among earlier studies, a large number of cross-sectional survey studies of short-term pandemics have been published. However, there are relatively few longitudinal studies over longer periods of time. In this perspective, recent studies have shown that longitudinal studies can contribute to the dynamic understanding of epidemics. The public’s overall psychological distress rose sharply early in the pandemic, before falling to pre-pandemic levels over the next few months [19]. The psychology of the elderly has also been affected by the long-term impact of the epidemic [20]. The psychology of ordinary people has also changed after the long-term development of the epidemic [21]. This kind of longitudinal research that emphasizes “temporal dynamic trends” is rare in previous studies [22]. Because of the discovery of these complex issues, it is currently unclear how the impact of epidemic policies will evolve over time, which may be related to specific influencing factors.

Due to the unpredictability of crises caused by sudden outbreaks, it is challenging to track the emotional changes of citizens during this period. In the early stages of the epidemic, the government adopted a cross-department tracking risk perception survey. This traditional survey method was small in scope, highly time-delayed, and expensive. The use of questionnaires in the study of emotional attitudes is a commonly used data collection method in this research direction. However, due to the limited amount of data, it is difficult to grasp the research issues in a macroscopic and in-depth manner. Using big data text mining methods, researchers can make a more macro-level summary of the impact of epidemic policies on public emotional attitudes and the influencing factors behind them. Currently, researchers have used this method to measure public reactions to government policies such as mental health and social prejudice. In terms of emotional attitude research, this method has also begun to be used to conduct related research. When big data mining is used to study emotions, attitudes and behaviors, due to the large sample size, its advantages are strong objectivity, high real-time performance, and significant effects. Therefore, online text analysis has become an important method for studying individual psychological characteristics and behavioral performance, and is more suitable for this study.

Based on these studies, we explore the emotional evolution of users in the sudden shift of government policies. This study has two main objectives: First, the emotional evolution process of Weibo users. The second is the changing pattern of influencing factors behind the emotional evolution of Weibo users. In order to study the emotional changes of social media users after the policy change, we obtained Weibo hot searches and online comments from November 1, 2022 to March 1, 2023 (from before the promulgation of China’s “New Ten” relaxed prevention and control policies 1 month to the time period when there are no hot searches related to “epidemic” on Weibo), and use machine learning algorithms to classify these online texts. The focus is on the peak (inflection point) changes in emotional evolution as the research object. Based on the existing research, this paper studies the following issues:

1. Analysis of the turning point of emotional evolution and “hot topic searches” events after the sudden opening-up policy.
2. Analysis of the evolution of emotional behavioral factors after the sudden opening-up policy.

## Data and method

**Fig. 1:**
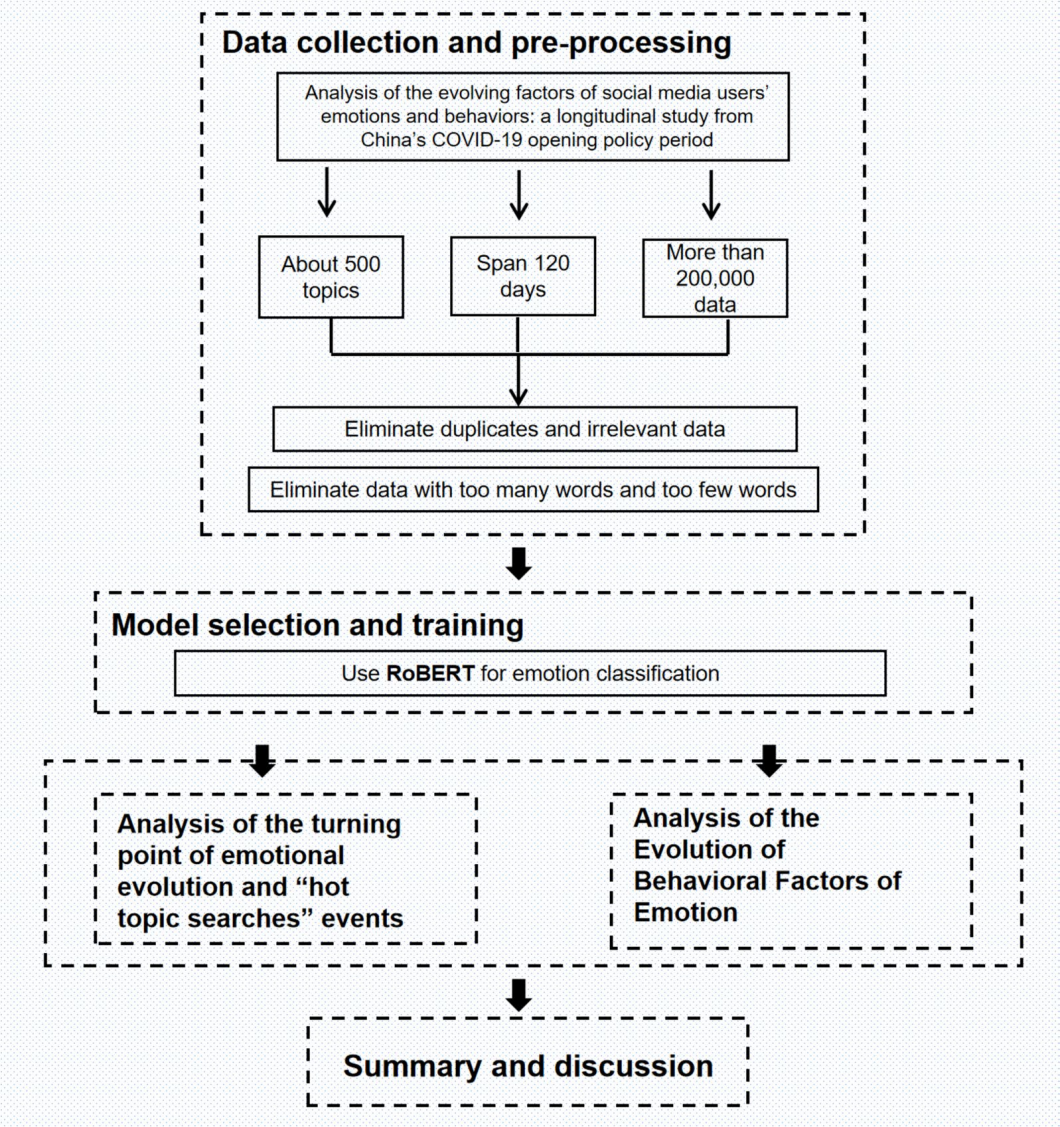
Research Framework of Emotional Evolution of Social Media Users

As shown in Fig. 1, the framework and methods used to analyze the emotional evolution of social media users in China after the sudden release of the COVID-19 epidemic is mainly divided into five parts: data collection and processing, model selection and training, analysis of the emotional evolution process, analysis of reasons for inflection points, and summary and discussion.

### Data collection and data preprocessing

In November 2022, the COVID-19 outbreak erupted in multiple regions of China, making it difficult to control. In December 2022, the Chinese government suddenly announced the complete lifting of epidemic control measures, leading to widespread infections and major universities announcing early holidays. In January and February 2023, people gradually recovered their health and began to enjoy the holidays, while also worrying about the possibility of another outbreak. With the resumption of work and school, the influenza virus began to spread among the population. During these four months, people’s emotional states underwent constant changes. We found the hotspots related to the epidemic based on the daily hot search list, and then used relevant Python technologies to collect data under the corresponding hotspots. We collected more than 200,000 pieces of data under nearly 500 hotspots from November 1, 2022 to February 28, 2023, each of which includes the text message posted by the user, the posting time, the number of likes, the number of reposts, and so on. We cleaned and organized the initial 200,000+ pieces of data, removed some duplicate content and content with more than 100 characters or less than 10 characters, and obtained 110,000 data after processing. We randomly selected 12,000 pieces of data for manual annotation, using “positive”, “negative”, and “neutral” to label the data.

**Fig. 2:**
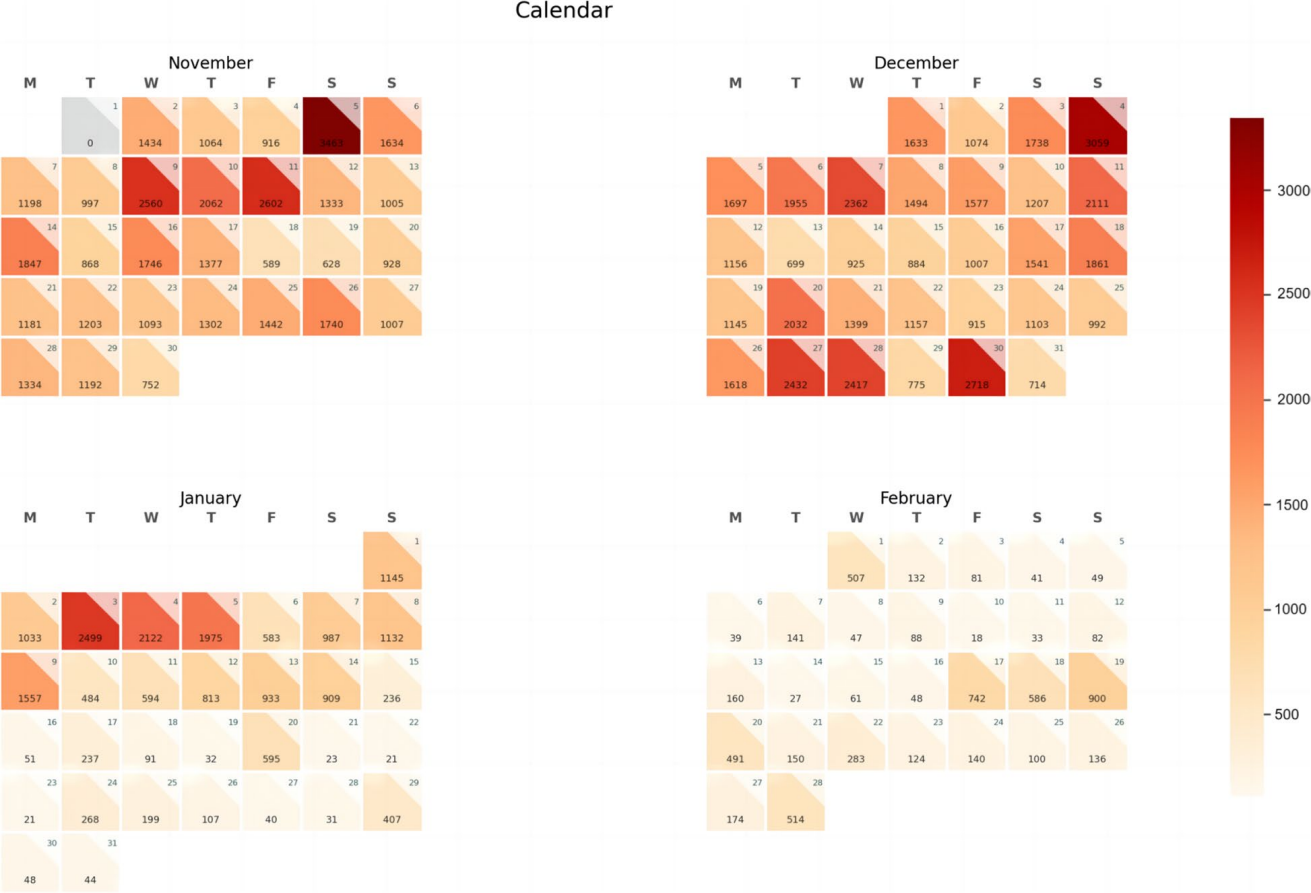
This is the data distribution calendar heatmap.(The number in each grid represents the amount of data for that day)

The following Fig. 2 shows the distribution of our processed data in a calendar heatmap, where we classified the data by publication time and analyzed the data for each day.

### Method

#### Sentiment classification

Sentiment classification refers to the process of categorizing text into positive or negative types based on the meaning and emotional information conveyed in the text. It is a way of categorizing the author’s bias, viewpoint, and attitude. With the rapid development of deep learning, algorithms based on deep learning have produced the current optimal results in many application fields, including computer vision, natural language processing, etc. Text sentiment classification is one of the many tasks in the field of natural language processing, which requires converting the words in the sentence into continuous real-valued vectors through word embedding to obtain input features. One commonly used word embedding algorithm is Word2Vec [23, 24], which learns word embedding vectors from text and then inputs them into CNN, RNN and other neural network models for sentiment feature learning. Recently, text sentiment classification methods based on pre-trained language models have begun to emerge. Large language models such as BERT [25] and GPT [26–28] series are first trained on a large amount of unlabeled text data through MLM tasks and next sentence prediction tasks, and then fine-tuned with a small amount of labeled text data in downstream tasks to obtain very good performance, greatly reducing the manual annotation cost of this article. We selected the Chinese pre-trained language model of RoBert and fine-tuned it with 12,000 annotated texts.

#### Factor analysis

In order to study the reasons for the emotional evolution of social media users, we need to analyze the influencing factors of different emotional states of users at different time periods. Firstly, we segmented the data of different emotional states at different time periods using “jieba”, an excellent Chinese word segmentation library that calculates the correlation probability between Chinese characters based on a Chinese word library and can group Chinese characters with high correlation probability into word groups. We filtered out some irrelevant word groups in the statistical word segmentation results, such as “so”, “this”, “those”, etc. Then, we counted the frequency of each word group and used it to construct a word cloud. We selected the words with higher frequency to obtain the main influencing factors for each emotion. We then calculated the proportion of each factor in the original data and summarized the key influencing factors that caused the emotional state during that time period.

## Discussion and analysis

**Fig. 3:**
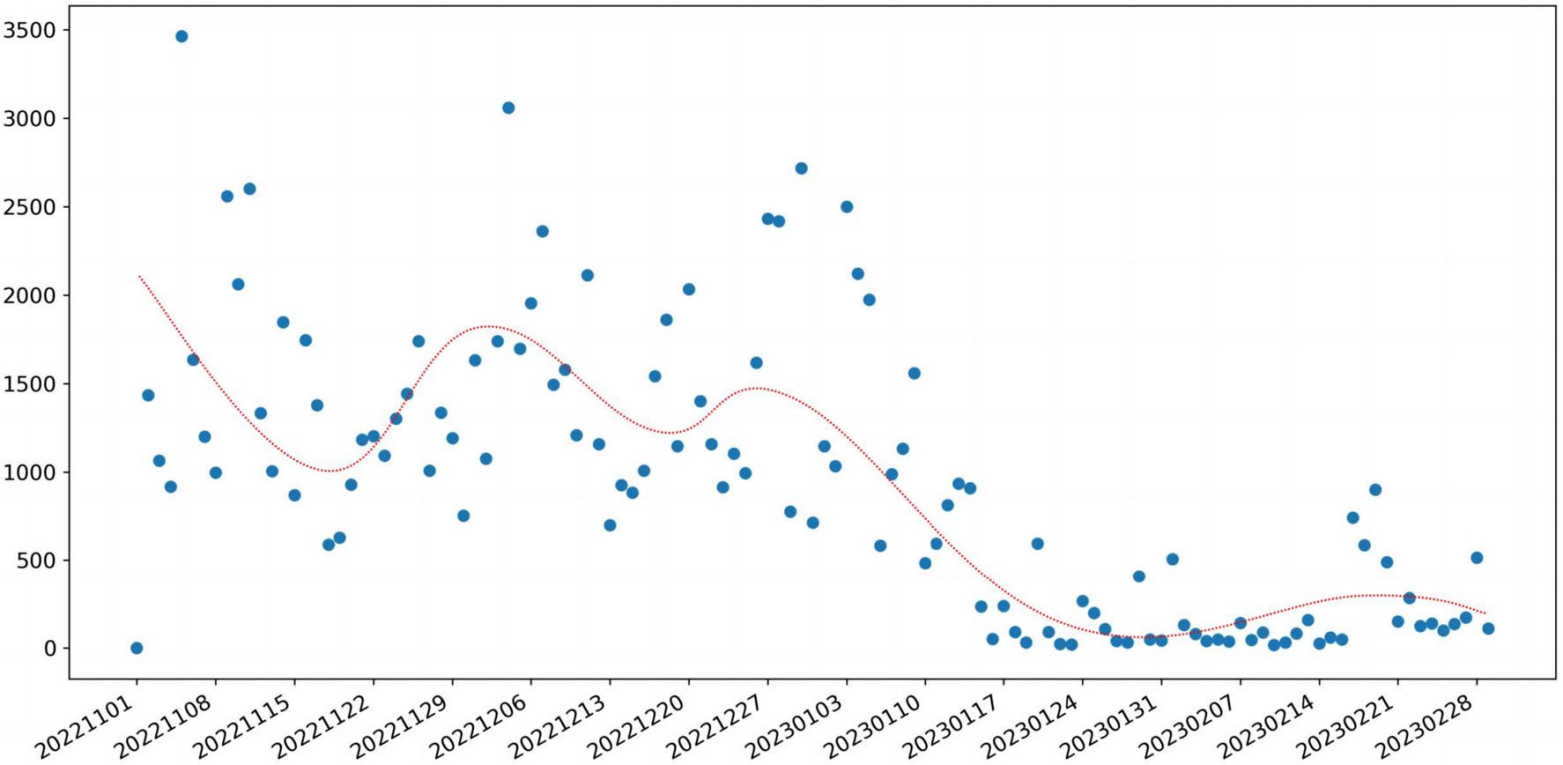
This is the daily number of Weibo posts in our dataset

We classified the data according to the order of user posting, dividing it into 120 days. The scatter Fig. 3 below shows the distribution of data volume for each day. From Fig. 3, it can be seen that from November 2022 to early January 2023, social media users had a high level of discussion on topics related to the epidemic. The discussion volume began to decline in mid-January 2023, but there was a slight increase in mid-February 2023.

## Analysis of the turning point of emotional evolution and “hot topic searches” events

### Analysis of the turning point in the evolution of positive emotions and “hot topic searches” events

**Fig. 4:**
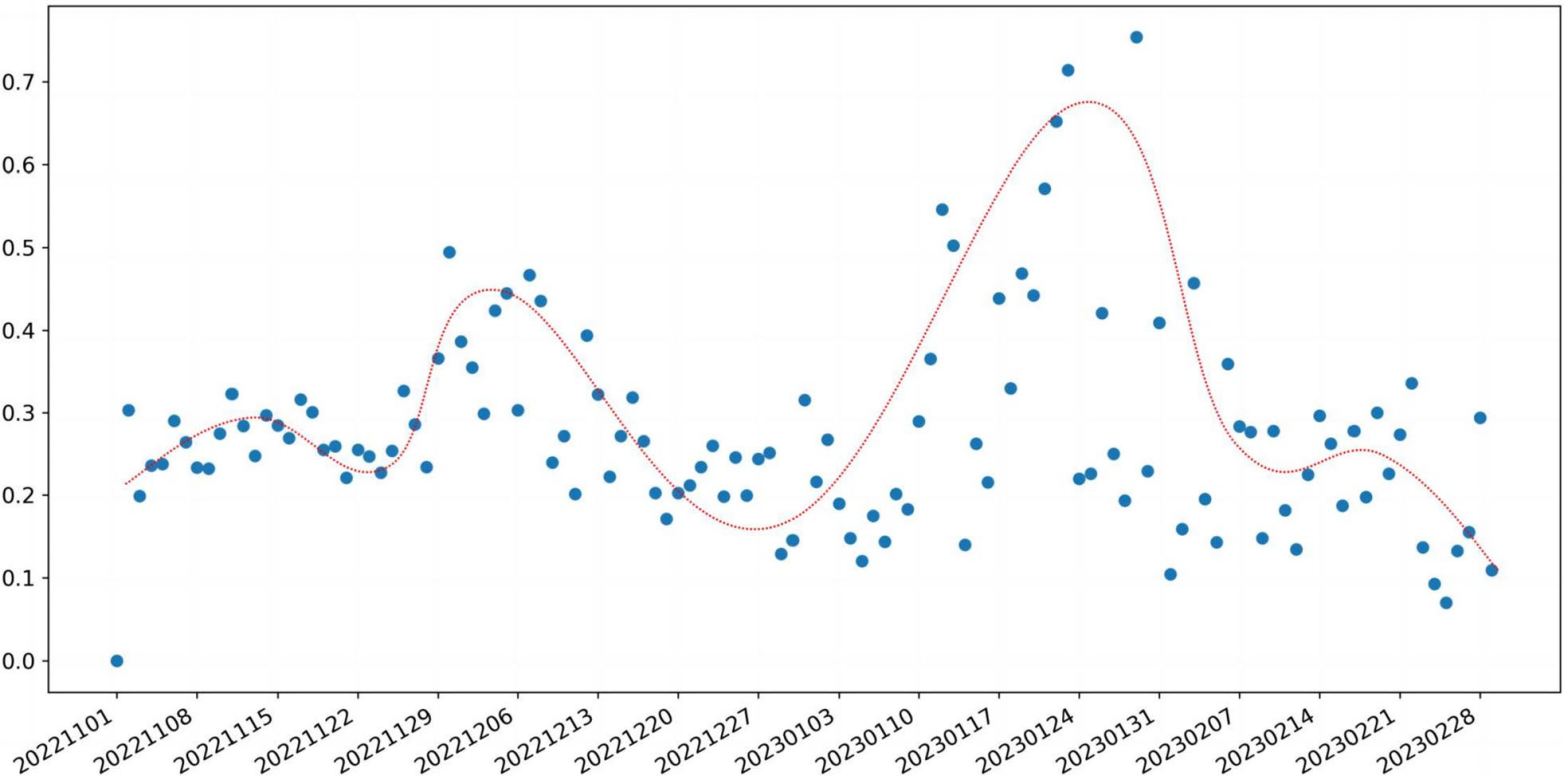
Scatter plot of daily proportion of positive emotions data (0.1-0.7 in the icon represents 10%-70%, which is the proportion value, and 1 represents 100%)

In order to analyze the process of positive emotional changes over time, we plotted a scatter plot of the distribution of the proportion of positive emotions over time and performed curve-fitting on the scatter plot, as shown in Fig. 4. From the Fig. 4, it can be seen that the curve of the proportion of positive emotions is relatively flat from early November 2022 to around November 23, 2022. It gradually increases starting from November 23, 2022, reaching its peak around December 6, 2022, and then begins to gradually decrease, reaching its minimum point around December 27, 2022. It then gradually increases again, reaching its second peak around January 25, 2023, before decreasing again. After a short period of flatness, it begins to decrease further from February 19, 2023.

In order to analyze the factors affecting the inflection points of the curve, we studied the Weibo hotspots on the day of the inflection points. The first turning point was around November 23, 2022. The hot topics at that time mainly included “ensuring that the 20 measures are true to form and not out of shape”^3^, “Shanghai further strengthens the health management of people coming and returning to Shanghai”, “Jinan epidemic prevention “Control”, “Zhengzhou will conduct 5-day mobility management”, “Guangzhou epidemic prevention and control”, “863 new local infections in Beijing”, “Nucleic acid testing volunteers were found to be positive by residents”, etc. The study found that these The hot topics of discussion are all related to the “strengthening of epidemic prevention and control and the increasingly serious epidemic situation.” The government’s epidemic control measures have been strengthened, but new cases have increased across the country. At this time, users began to question the “strict blocking policy”, which triggered a chain of events that quickly spread and became a hot topic on Weibo, causing users’ positive emotions to reach a low point. The event of concern at the positive emotional trough is the “blocking policy”.

The second turning point was around December 6, 2022. The hot spots at that time mainly included “Ten New Measures for Epidemic Prevention and Control”, “Sanya Air Ticket and Hotel Bookings Soared Three Times”, “The number of medical doctors did not increase significantly after the adjustment of Guangzhou’s epidemic policy”, “There are new optimizations in prevention and control measures”, “We are officially on the road to ending the epidemic”, etc. These hot topics of discussion are all related to the adjustment of epidemic policies. At this time, the Chinese government suddenly announced the relaxation of epidemic control, marking China’s 3 The strict epidemic control policies have ended in 2019, and people’s lifestyles are about to resume. The essential reason is the weakening of the government’s epidemic control measures. The user’s positive emotions reach a peak. The events of concern for users’ positive emotional peaks are “Opening policy” and “Travel”.

The third turning point was around December 27, 2022. The hot spots at that time mainly included “Qingdao currently has about 500,000 new infections every day”, “New coronavirus strains have been detected in Shanghai”, and “Why are there secondary infections?” , “Be wary of viral conjunctivitis after becoming infected”, “Will infection with COVID-19 affect memory”, “A woman has not left home for more than 20 days for fear of being infected”, etc. These hot topics of discussion are all related to “symptoms of COVID-19” and “secondary infection” Relevantly, at this time, most people have experienced and recovered from the new coronavirus infection, and are fearing the sequelae and secondary infection. The user’s focus at this time is his or her own illness and recovery, which is an expression of personal issues. Positive emotions reach a minimum. The focus of users’ positive emotional valleys begins to change to “sickness symptoms”.

The fourth turning point was around January 24, 2023. The hot spots at that time mainly included “Q &A on 4 hot spots for epidemic prevention during the Spring Festival”, “How uninfected people can protect themselves”, etc. At this time, the epidemic is basically over, and it is approaching the Chinese “Spring Festival”^4^, people discussed how to protect themselves during the festival and began to enjoy the joy brought by the festival. At this time, the user’s focus is on enjoying the festive atmosphere, which is a social issue. The user’s positive emotions once again reached a maximum value. The events with peak positive emotions among users are “celebration of festivals” and “personal epidemic protection”.

In short, the “hot topic searches” events for the evolution of users’ positive emotions include: intensification of the epidemic (low positive emotions) - changes in lockdown policies (high positive emotions) - illness (low positive emotions) - recovery, holidays (high positive emotion) - end of vacation, return to work, back to school infection (low positive emotion). Therefore, we found that events that affect positive emotions are mainly related to “epidemic policies” and “holidays”. Generally speaking, the events of concern for the rise and fall of positive emotions are “sickness” and “sickness again.” Events of concern with elevated positive emotions are “changes in lockdown policies” and “holidays”.

#### Analysis of the turning point in the evolution of negative emotions and “hot topic searches” events

**Fig. 5:**
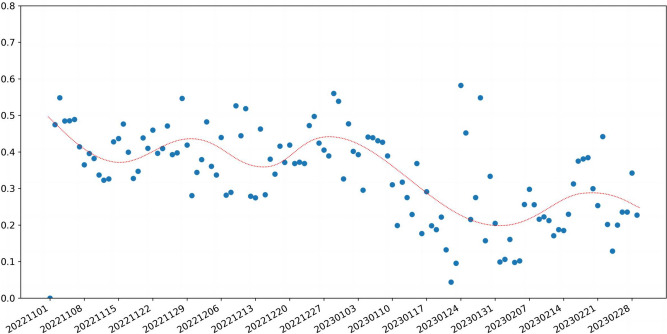
Scatter plot of the proportion of daily negative emotions (0.1-0.8 in the icon represents 10%-80%, which is the proportion value, and 1 represents 100%)

Similarly, in order to analyze the process of negative sentiment changing over time, we also plotted a scatter plot of the distribution of the proportion of negative sentiment over time and fitted a curve to the scatter plot, as shown in Fig. 5.

From the Fig. 5, it can be seen that the proportion of negative sentiment remained relatively flat from early November 2022 to around December 26, 2022, and then gradually decreased. It reached a minimum point around January 30, 2023, and then began to gradually increase, reaching a maximum point around February 18, 2023, before gradually leveling off.

We conducted a study on the Weibo hot topics on the days of the above inflection points. The first turning point is around December 26, 2022. The main hot spots include “the boy who was positive for 5 days had no fever and suddenly convulsed non-stop”, “be careful of viral myocarditis after getting sick”, “experts responded to whether Omicron will cause white blood.” Pulmonary Phenomenon”, “6 Danger Signs of Myocarditis”, etc. Research has found that these discussion hot spots are all related to the symptoms and sequelae of the epidemic. At this time, some people have experienced the new coronavirus infection and recovered, and are discussing the impact of the sequelae. There are also some people who are infected with the new coronavirus and are suffering from the disease. The proportion of users’ negative emotions has always been at a high level. Users’ focus at this time is on “disease treatment” and “second infection”. These are related to events of concern related to “illness”. At this stage, the peak negative emotional concern event for users is “COVID-19 disease”.

The second turning point is around January 30, 2023. The hot spots mainly include “Q&A on 4 hot spots for epidemic prevention during the Spring Festival”, “How can uninfected people take personal protection”, etc. At this time, the epidemic is basically over, and people begin to discuss how to do a good job. During the holidays, personal protection is required, and the joy brought by the holidays is enjoyed, and the proportion of users’ negative emotions reaches a minimum. At this time, most users have “recovered from illness”, and events related to “holidays” are their focus.

The third turning point occurs around February 18, 2023. The hotspot is mainly related to the end of the holiday. Users need to return to the city where they work through public transportation, and they are worried about “second infection” of the new crown on the way. The proportion of negative emotions reaches a maximum value. The “festival” is over, and users are paying more attention to events related to the “second infection”.

In short, the evolution of users’ negative emotions and public opinion events include: illness (high negative emotions) - festivals (low negative emotions) - secondary infection (high negative emotions). Therefore, we found that public opinion events that affect negative emotions are mainly related to “sickness.” Generally speaking, the types of public opinion events that increase negative emotions are “sickness” and “sickness again.” The type of public opinion event with decreased negative sentiment is “festival”.

### Analysis of factors influencing emotional evolution

In order to further analyze the factors influencing the evolution of the epidemic, we conducted separate analyses of data on positive emotions and negative emotions, identified some of the more frequent influencing factors, and analyzed the temporal evolution of these factors.

#### Analysis of positive emotional factors

**Fig. 6:**
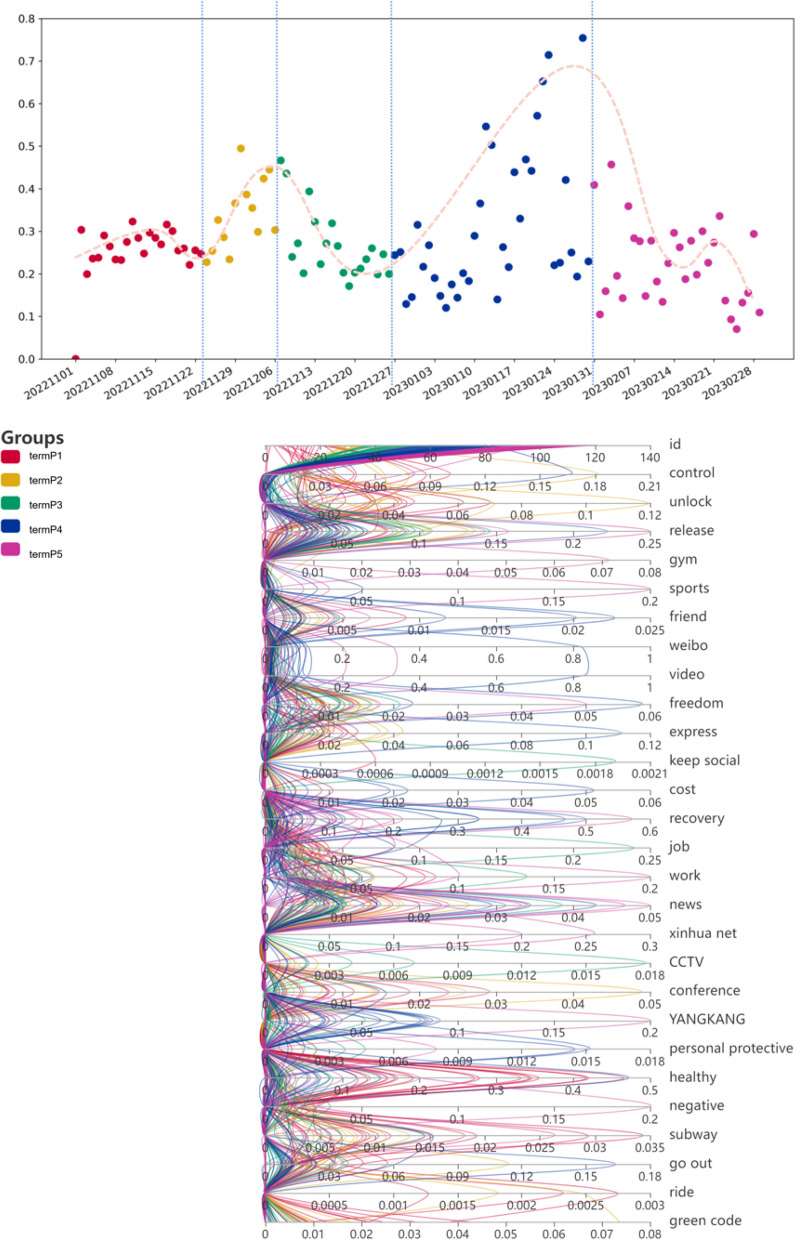
Analysis of positive emotional factors.(1.we use ids from 1 to 120 to represent the dates from November 1, 2022 to February 28, 2023. Each time period is represented by a different color.2.TermP1: 2022.11.01-2022.11.23; TermP2: 2022.11.24-2022.12.07; TermP3: 2022.12.08-2022.12.26; TermP4: 2022.12.27-2023.01.30; TermP5: 2023.01.31- 2023.02. 28.3. Each color represents each corresponding time period. 4. The value in the icon data represents a percentage. For example: 0.21 in “Control” represents 21%; the yellow 0.18 means that during the TermP2 time period, “Control” accounts for The highest ratio is 18%)

In the analysis of positive emotional factors, we selected control, let go, unlock, the gym, movement, friend, Weibo, video, freedom, express, keep social, cost, recovery, work, job, news, the Xinhua, CCTV network, conference, YangKang, personal protective, healthy, negative, subway, go out, ride, and green code as the main influencing factors. We analyzed the proportion of each influencing factor in all positive emotional data for each day, as shown in Fig. 6.

In the figure, we use ids from 1 to 120 to represent the dates from November 1, 2022 to February 28, 2023. Each time period is represented by a different color. It can be seen that positive emotion low value - high value: the time period from TermP1 to TermP2. In TermP1, the factors “control, unlock, release, keep social, healthy, subway, and ride” account for the highest proportion, and users’ positive emotions are at their lowest during this period. Then, the government announced the lifting of China’s epidemic control policy, and “control, unlock, express, conference, go out, green code” in TermP2 became the main factors. Users’ positive emotions are at their highest during this period. Due to the adjustment of epidemic policies, users’ emotional evolution has changed, and behavioral factors have also changed. User behavior has evolved from “epidemic policy control” to “travel and media”.

Positive emotion high value - low value: time period from TermP2 to TermP3.In TermP3, the factors “keep social, job, work, CCTV” account for the highest proportion. Users are worried that after the epidemic policy is relaxed, they need to go to the company to work, and there will be crowds of people, causing company employees to infect themselves with the new coronavirus, causing panic, and users’ positive emotions quickly fell to the lowest value. Behavioral expression of users’ positive emotions: evolving from “travel and media” to “work”.

Positive emotion low value - high value: the time period from TermP3 to TermP4. In TermP4, the factors “control, release,,friend,WeiBo,news,video,express,cost,recovery,YANGKANG,personal protective,healthy,freedom,go out” account for the highest proportion. . Due to the large-scale infection of new coronavirus patients in a short period of time, they began to gradually recover. At the same time, China has entered into the most important holiday atmosphere of the Spring Festival. Users’ focus on positive emotional factors began to diversify and gradually returned to daily life. Therefore, the positive emotions at this time enter the highest value. Behavioral expression of users’ positive emotions: from “work” to “open policy, friend gatherings, media, economic recovery, personal recovery” and other aspects.

Positive emotion high value - low value: the time period from TermP4 to termP5. In TermP5, the factors “relase, gym, sports, WeiBo, video, freedom, recovery, work, news, xinhua net, YANGKANG, negative” account for the highest proportion. The Spring Festival is over, users need to return to work, and there is a large-scale migration of the population [29]. What they are about to face is the secondary infection of the new crown. Therefore, users focus on information released by the media about the resurgence of the epidemic, their own sports health, personal recovery, and prevention of the sequelae of the new coronavirus. Behavioral expressions of users’ positive emotions: from “open policy, friend gatherings, government media, economic recovery, personal recovery” to “media, personal recovery, secondary infection”.

**Fig. 7:**
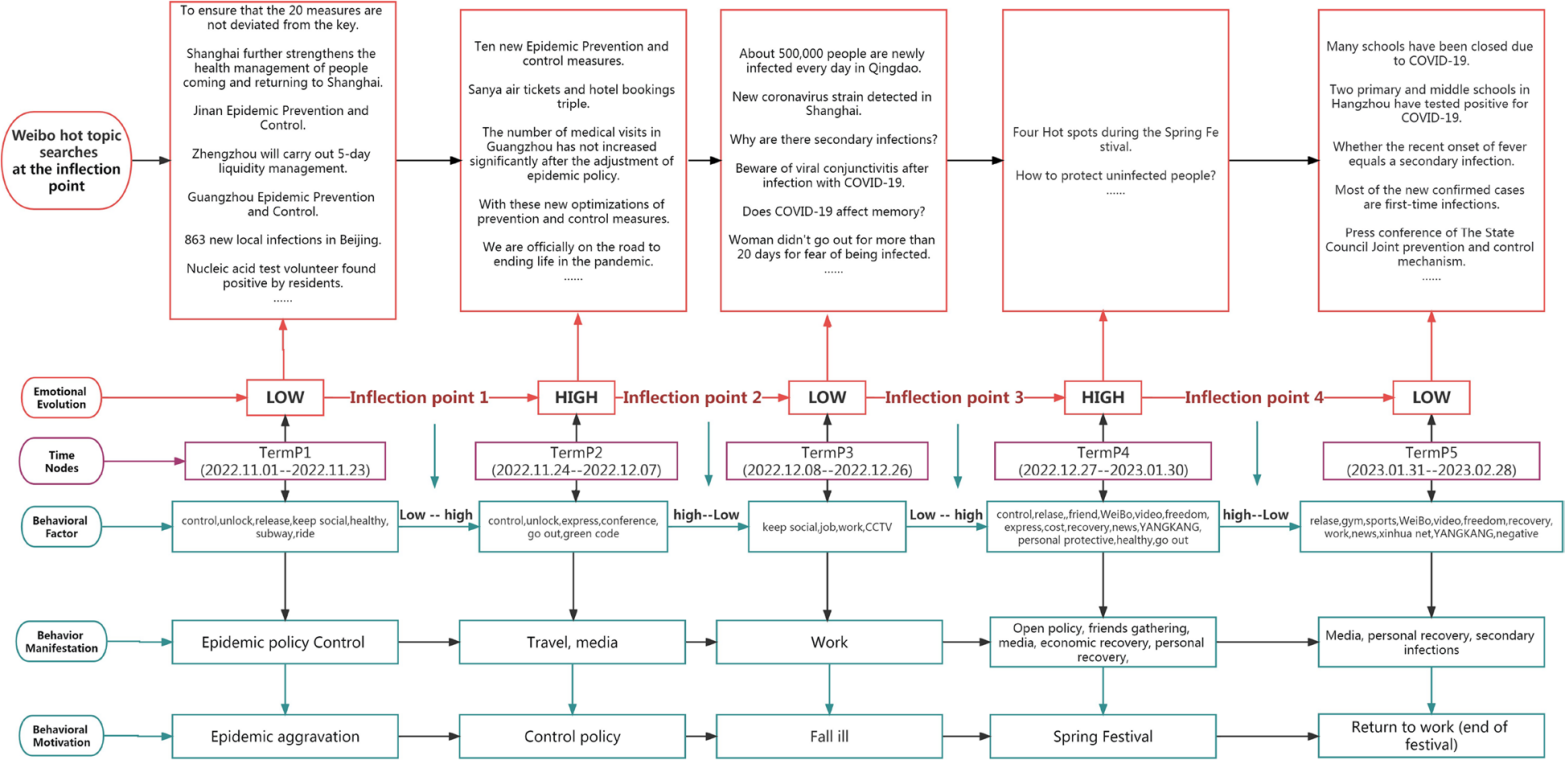
Analysis and summary of the evolution of positive emotions

Figure 7 in short, we found that the behavioral factors of social media users’ positive emotions change with the adjustment of epidemic policies and the development of epidemic infections, reflecting the evolution of behaviors: “epidemic policy control” - “travel, media” - “work” - - “Open-door policy, gatherings of friends, media, economic recovery, personal recovery, etc.” - “Media, personal recovery, secondary infection.” Motivations affecting the evolution of behavioral factors: worsening of the epidemic - control policies - illness - celebrating festivals - returning to work (end of festivals).

#### Analysis of negative emotional factors

**Fig. 8:**
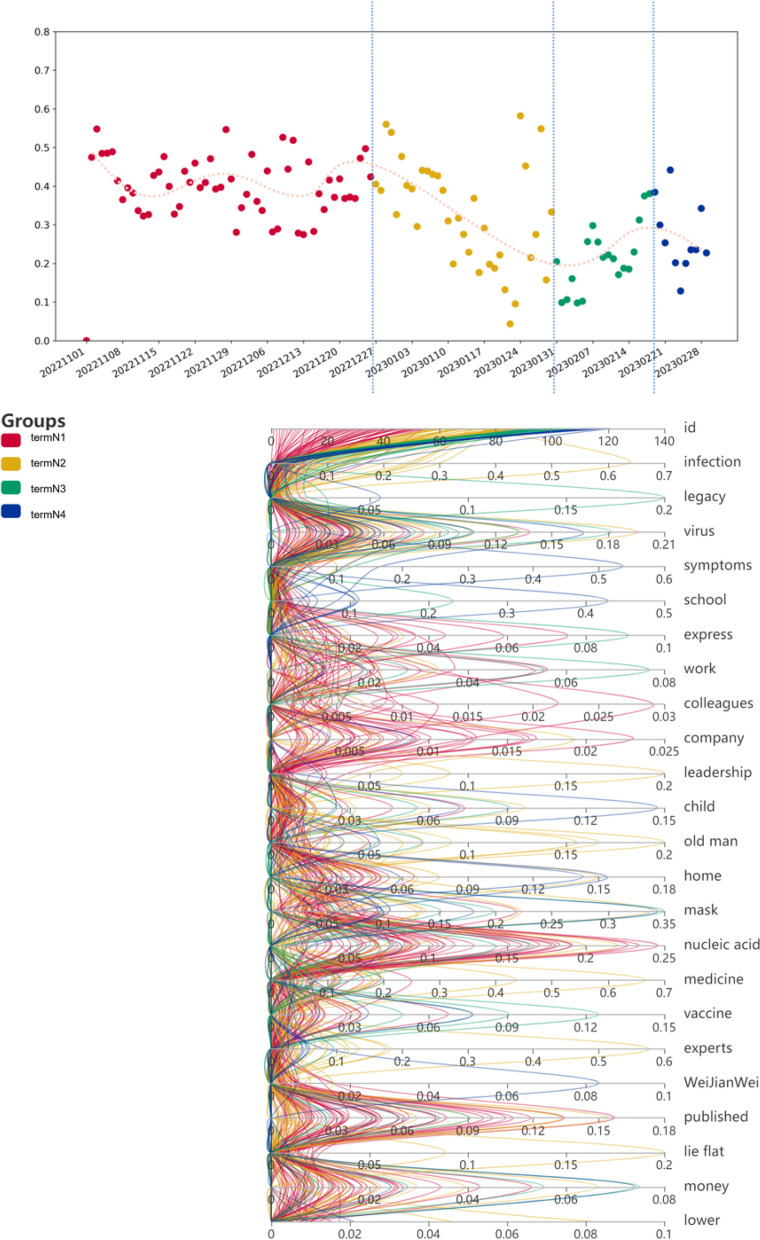
Analysis of Negative Emotional Factors.(1.we use ids from 1 to 120 to represent the dates from November 1, 2022 to February 28, 2023. Each time period is represented by a different color.2.TermN1: 2022.11.01-2022.12.26; TermN2: 2022.12 .27-2023.01.30; TermN3: 2023.01.31-2023.02.18; TermN4: 2022.02.18-2023.02.28.3. Each color represents each corresponding time period. 4. The values in the icon data represent percentages, for example: The 0.2 in “legacy” represents 20%; the green 0.2 means that during the TermN3 time period, “legacy” accounted for up to 20%)

In the analysis of negative emotions data, we selected infection, virus, legacy, symptoms, school, express, work, colleagues, the company, leadership, child, the old man, home, mask, the nucleic acid, medicine, vaccine, experts, WeiJianWei^5^, published, lie flat, money, and lower as the main influencing factors, and analyzed the proportion of each influencing factor in all negative emotion data for each day, as shown in Fig. 8.

High value - low value of negative emotion: the time period from TermN1 to termN2. In termN1, the factor “published, necleic acid, express, colleagues, company” has the highest proportion, and the user’s positive emotions during this time period are the lowest. During this period, 1. The Chinese government issued the “New Ten Epidemic Prevention Policies”, completely lifting the three-year epidemic containment policy. 2. When the epidemic breaks out on a large scale, most people need to undergo nucleic acid testing every day. 3. “Express” has become an important source of infection for the new coronavirus epidemic. 4. Because the lockdown policy is lifted, people need to go to work normally, which will cause people to gather and accelerate the infection of the epidemic. It mainly includes three aspects of behavioral performance: policy, illness, and going to work (fear: worried about being infected with the new crown). Then, the peak of the nationwide large-scale epidemic infection in termN1 ended with the peak of termN2. In termN2, “infection, virus, company, leadership, child, old man, home, mask, nucleic acid, medicine, experts, published, lie flat, lower” has become the main factor. Users’ negative emotions are at their lowest during this period. Mainly included behavioral manifestations: family, experts, illness, prevention, work, and infection rate reduction. Most of the COVID-19 patients recover from illness, and with the Chinese Spring Festival approaching, users are paying more attention to holiday enjoyment. The emotional evolution of users has shifted, and behavioral factors have also changed. The behavioral influencing factors of users’ negative emotions have evolved from “illness” to “recovery, work (neutral: recovery and return to work), and family”.

Negative emotion low value - high value: the time period from termN2 to termN3. In termN3, “legacy, virus, express, work, mask, vaccine, money” has become the main factor. Mainly included behavioral manifestations: illness, sequelae, prevention, work (unable to make money). Negative emotions are elevated during this time. After the festival, users need to leave their hometown and return to the city to continue working, and may even suffer “COVID-19 discrimination” [30]. They began to worry that clusters of infections (mainly second infections) would occur during the return journey, which would lead to sequelae and even affect income. The behavioral influencing factors of users’ negative emotions develop from “recovery, work (neutral: recovery and return to work), family” to “illness, sequelae, prevention, work (unable to make money)”.

High value - low value of negative emotion: the time period from termN3 to termN4. In termN4, “symptoms, school, mask, WeiJianWei, money” has become the main factor. Mainly included behavioral manifestations: schooling, prevention, and economy. Negative emotions decrease during this period. This stage is mainly the time when students start school. Users are more concerned about outbreaks when children gather at school and pay more attention to preventing infection. At the same time, after the holidays, people have returned to their jobs and the economy has begun to improve. The behavioral influencing factors of users’ negative emotions have evolved from “illness, sequelae, prevention, and work (unable to make money)” to “schooling, prevention, and economy.”

**Fig. 9:**
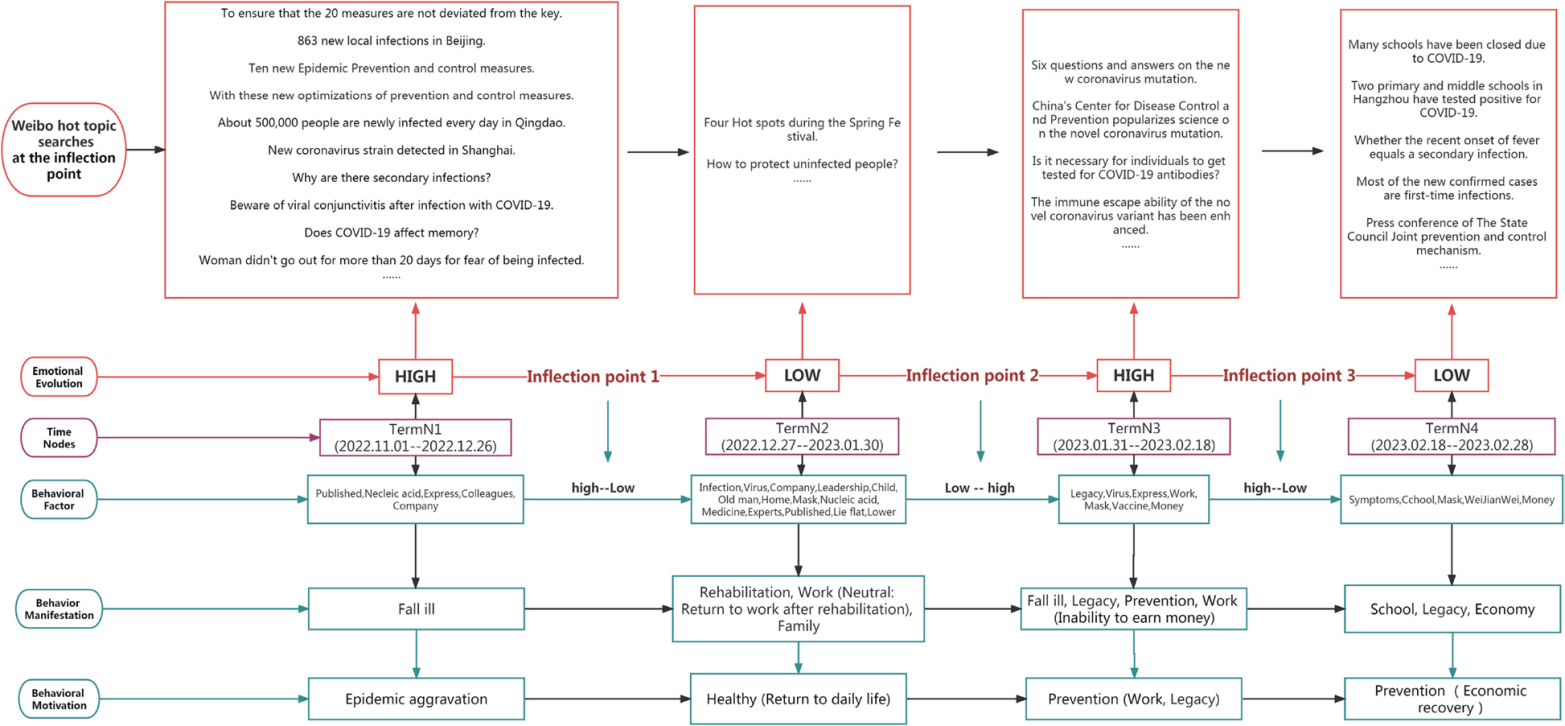
Analysis and summary of the evolution of negative emotions

In short, we found that the behavioral factors of social media users’ negative emotions change with the development of epidemic infection, and the evolution of behavioral behaviors: “sickness” - “recovery, work (neutral: recovery and return to work), family” - “Sickness, sequelae, prevention, work (cannot earn money)” - “schooling, prevention, economy”. Motives affecting the evolution of behavioral factors: worsening of the epidemic - recovery (returning to daily life) - prevention (work, sequelae) - prevention (schooling), and economic recovery (Fig. 9).

## Discussion

The Online media is an important channel for the government to announce epidemic containment policies to citizens. It is also the fastest and most direct way for citizens to feedback relevant information. In the field of public health, sentiment analysis has been widely used in research in recent years to provide a more comprehensive and effective understanding of the way the media conveys COVID-19 policies to the public and the public’s emotional response [31, 32]. This study collected data from November 1, 2022 to February 28, 2023, a total of 120 days, which includes the strict control stage before the implementation of the open policy, the sudden implementation of the open policy stage, the nationwide rapid infection stage during the Chinese epidemic, and the most important Spring Festival holiday stage during the epidemic infection process in China. Collecting data during this time period will help to comprehensively understand the emotional changes and behavioral factors of social media users under the large-scale epidemic infection in China within a short period of time. Conducting research in the form of “dynamic time evolution” has special research significance. This includes 500 Weibo hotspot searches and 200,000+ comment data. The collected data is analyzed through deep learning algorithms. The main results and discussions for each research question are as follows.

### Analysis of the turning point in the emotional evolution of social media users and “hot topic searches” events

We use the trained model to analyze the data, calculate the emotional proportion of social media users every day, and discuss the inflection point of emotional evolution and “hot topic searches” events through the development of the timeline. This method allows us to understand the reasons for the emotional turning point after the sudden opening-up policy and find the events of concern that trigger emotional changes.

Different from previous research conclusions on the single development of emotional changes [16], we found that the positive emotions and negative emotions of social media users affected by China’s sudden opening-up policy are different in the inflection point of emotional evolution and the performance of events of concern.

Negative emotions are relatively stable and gradually weaken as the epidemic develops [33]. Three major emotional evolution turning points were formed. The focus of events that affect negative affective fluctuations are “sickness”, “second infection” and “holidays”. The influencing factors of negative emotions are relatively single. Mainly factors directly related to the “epidemic” such as “illness”, “second infection”, and “sequelae”. Therefore, when considering formulating unblocking policies, you need to pay attention to the formulation of “sickness”-related policies and response plans, which can help quickly reduce users’ negative emotions. Among them, the events of “second infection” and “sequelae” are important contents that continue to affect negative emotions. Therefore, more attention should be paid to the behavior of users with negative emotions.

From the perspective of positive emotions, the positive emotions of social media users fluctuate greatly and are easily affected by factors such as the social environment and the development of epidemic policies. Four major emotional evolution turning points were formed. From the strict implementation of the new crown lockdown policy to the sudden promulgation of the opening policy. Users directly faced the short-term and large-scale outbreak of the COVID-19 epidemic in China, and their emotions were affected by multiple factors such as “government”, “individual” and “society”. Among them, we found that positive emotions are a comprehensive response to multiple factors, such as: users’ concerns about the “intensification of the epidemic”, carnival about the “opening policy”, fear of personal “sickness”, yearning for “festivals”, etc. . This is the focus of the development of the epidemic event, and the process in which users change according to their own needs. In the face of public health emergencies, users have been in a negative emotional state, are more sensitive to various events, and express greater emotional fluctuations. However, “epidemic intensification”, “opening policy”, “sickness”, “holidays”, etc. are events that need to be paid attention to before formulating epidemic unblocking policies. These are the concerns that affect the behavior of positive emotional users.

### Analysis on the evolution of behavioral factors of social media users’ emotions

Due to the sudden implementation of China’s government open policy, the outbreak of the epidemic has spread rapidly. Therefore, the influencing factors behind the emotional evolution of social media users are an important reference for the government’s subsequent decisions. Although the thematic emotional changes of users do not vary significantly in different stages of development, the underlying influencing factors can undergo significant changes [34]. The study of such changes can significantly assist in the dynamic disclosure of government decisions.

Based on the four turning points in the evolution of positive emotions, we divide the development time of positive emotions into five stages. They are “TermP1-TermP5” respectively. The positive emotional behavior of social media users continues to change as the epidemic develops. The evolution of behavioral manifestations is “Epidemic policy Control-Travel, Media-Work-Open policy, Friends gathering, Media, Economic recovery-Media, Personal recovery, Secondary infections.” The motivations for the evolution of these behaviors are the gradual intensification of the epidemic, the lifting of the lockdown policy, large-scale illness, celebrating the Spring Festival, and returning to work in the city (the end of the festival). In addition to changes caused by government policies (the lifting of lockdown policies), users’ positive emotions also include personal health and festivals. In the face of epidemic policy adjustments, the recovery of positive emotions is short-lived. The government needs to continue to pay attention to personal health-related issues in the future and alleviate the harm of the disease to individuals. At the same time, using the joyful atmosphere of the festival to shift the focus of the epidemic to holiday celebrations will help improve users’ positive emotions.

Based on the three turning points in the evolution of negative emotions, we divide the development time of positive emotions into four stages. They are “TermN1-TermN4” respectively. The evolution of behavioral manifestations is “Fall ill–Rehabilitation, Work (Neutral: Return to work after rehabilitation), Family–Fall ill, Legacy, Prevention, Work (Inability to earn money)–School, Legacy, Economy”. The motivations for these evolving behaviors are to intensify the epidemic, restore health, and prevent secondary infections. Among them, the behaviors of “preventing second infection” are slightly different, one is Work, Legacy, and the other is Economic recovery. In the face of negative emotions, users’ emotional behaviors mainly focus on illness and sequelae, and economic income. After the government adjusts its epidemic policies, it needs to pay attention to psychological counseling for large-scale infected people. Sickness is inevitable. Although most of the recovered people have not yet been “infected for the second time”, their fear has begun to spread, and they even believe that even if the lockdown policy is lifted, their economic income will still be affected. Changes in mentality will affect financial income [35]. Policies after recovery from illness should pay more attention to the impact of “second infection and sequelae”.

In short, China’s population of 1.4 billion has implemented strict containment policies for three years, but the outbreak has been difficult to control. Prolonged lockdowns have less impact on the control of Covid-19 [36]. The government adopted the lockdown policy to lift the epidemic and faced large-scale epidemic infections. Sudden policy shifts, users’ emotions change with the development of the epidemic situation.

Different from previous studies [16, 17, 37], in addition to mainly solving the problem of “disease treatment”, the government also needs to continue to pay attention to personal health and provide guidance for the recovery of personal health. At the same time, use festivals (not holidays) to guide and exaggerate the festive atmosphere and ease the tension of the epidemic. The time point is also very important. The festival should be about one month after the large-scale epidemic is over. Because at this point in time, the user has basically recovered, but the negative emotions are still high. It requires both personal health recovery guidance and reduction of negative emotions. There is also a need to celebrate festivals and exaggerate positive emotions. This can quickly alleviate users’ negative emotions and contribute to social stability and economic recovery.

## Conclusion

The COVID-19 pandemic is one of the most far-reaching events globally in recent years. The Chinese government’s strict prevention and control policies have provided strong guarantees for China’s economic development and citizens’ health and safety. However, the sudden shift in China’s government prevention and control policies in early December 2022 posed severe challenges to the Chinese government and citizens. Online comments from social media users recorded the impact of the pandemic prevention and control policy changes on society from different perspectives. To study the impact of government pandemic management measures on citizens during this special period, we continued to collect data on China’s Weibo for 120 days from November 1, 2022 to March 1, 2023, including 500 Weibo hotspot searches and 200,000+ comment data. And through the sentiment analysis of deep learning algorithms, we study the emotional development turning points of social media users, related “hot topic searches” events, and the evolution of behavioral factors. Through a dynamic study of the evolution of emotions and behaviors of social media users over a four-month period, it provides a valuable perspective for related research in China and other countries and regions.

Compared with the results of previous studies, we draw the following conclusions.

First, after the Opening policy is released, users’ positive and negative emotions fluctuate [15]. Previous research has shown that citizens have positive emotions towards the lifting of lockdown policies. Some research reports point out that negative emotions have an important impact on public panic [38], and focus on the spread of negative emotions on social media. This study found that the evolution of positive emotions plays an important role in reducing the negative impact of the epidemic. The evolution of negative emotions is relatively stable and will continue to decline with the implementation of government measures. However, for positive emotions with large fluctuations, more attention should be paid, and continuous attention should be paid to the dynamic development of behavioral elements of positive emotions, which can help quickly reduce users’ negative emotions.

Second, the evolution of behavioral elements deserves more attention. Studies have pointed out that the key issue is to balance saving lives and saving people’s livelihood, and health and economic anxiety are important considerations during the new crown epidemic [39]. This study found that in the long-term development of the policy, continuous attention to: disease treatment, personal health recovery, festival atmosphere, and reduction of publicity on the harm of “new crown sequelae and second infections” are the factors that affect users’ emotions. Changing behavioral concerns. As time goes by, dynamically changing the focus of behavioral elements will help alleviate social anxiety about the impact and stabilize social development.

Third, it is necessary to change the “hot topic searches” event by guiding the user’s behavioral focus to control the inflection point of the user’s emotion. “Hot topic searches” will affect the propagation path of Weibo topics [40]. In this study, we found that users’ behavioral elements are closely related to “hot topic searches” events, and the chain reaction of events reflects the inflection point of users’ emotions. There are big differences in the behavioral elements, behavioral expressions, and behavioral motivations of positive emotions and negative emotions. In the process of policy implementation, the government should pay attention to changes in keywords that are elements of user behavior, predict events where users’ emotions change, continue to pay attention to events and formulate plans.

The main contributions of this article include: First, we reveal for the first time the inflection point of the emotional evolution of social media users, “hot topic searches” events and behavioral evolution of Opening policy. During the implementation of China’s sudden liberalization policy, the positive emotions of social media users were divided into 4 inflection points and 5 time periods, and the negative emotions were divided into 3 inflection points and 4 time periods. Behavioral factors are different at each stage of each emotion. And the evolution patterns of positive emotions and negative emotions are also different. Among them, negative emotions decrease steadily; positive emotions are highly sensitive and prone to obvious fluctuations. Second, there are significant differences in the evolutionary manifestations and behavioral motivations of negative emotions and positive emotions. Previous research has paid more attention to negative emotions [38]. We can try to guide behavioral motivations and behavioral expressions to weaken the negative emotions caused by negative emotions. Third, in the context of open policy research, longitudinal research from the perspective of time series development is more important. Previous studies have focused on the “Epidemic lockdown policy” [10–12], some discussing socioeconomic factors as a whole, and some discussing the contradictions of support or opposition [17]. Our research pays more attention to the lasting impact of sudden opening policies. Study the inflection point of emotional evolution and “hot topic searches” during the policy implementation process, and discuss the evolution of social media users’ emotional behavioral factors from the “emergency policy” event ontology to more intuitively and dynamically reflect China’s epidemic outbreak The impact of open policy on society. Long-term sentiment data evolution analysis also helps us examine our social mechanisms and social preparedness in the face of disasters such as the COVID-19 pandemic, and improve the government and public’s prevention capabilities against such events. Fourth, this study provides directions and ideas for sustained policy propaganda work. China responded to this policy shift without adequate publicity preparations. As a result, the design of publicity and information on relevant content cannot meet the actual needs. This study can provide a reference for similar situations in the future, and formulate publicity and design in advance, which can help quickly alleviate the impact of relevant policies on society. In conclusion, traditional survey methods are limited to individual responses to the COVID-19 pandemic [41], and less involved in the extraction and summary of emotional influencing factors. Based on social media text data mining and dynamic evolution research with time series, it is more timely and accurate than overall static research [42]. Emergency management departments of the government can design propaganda content and answer doubts in advance to alleviate the negative impact of events. The sudden implementation of China’s COVID-19 relaxation policy provides a meaningful research object for this study. From a global perspective, only China has experienced this situation, making it more valuable for research.

There are also certain limitations to this study. Firstly, we mainly discuss China’s sudden relaxation policy, which may not have the general characteristics of research on other countries’ open policies. Secondly, we only collected user information from Weibo, and data from emerging video apps such as Tik Tok may also have certain representativeness. Thirdly, we used deep learning sentiment analysis algorithms to automatically identify the emotional expressions of each text unit, despite extensive manual labeling and training. However, due to the common phenomenon of Chinese polysemy, some emotional information in the document may not be accurately expressed.

For future research in this field, we believe that although the World Health Organization (WHO) announced the end of the global COVID-19 emergency on May 5, 2023^6^, the actual pandemic crisis has not ended, and related hot searches on “COVID-19 second transmission in China” reappeared on social media platforms on May 15. Therefore, continuous dynamic research on social media users’ emotional changes is more valuable than ever. In future research, more attention needs to be paid to the influencing factors behind emotions and corresponding measures. This will help relevant propaganda departments design information images and video content to quickly inform citizens of relevant policy implementations. This will not be a static measure issuance, but a real-time dynamic measure update system and propaganda system. This may be one of the research directions in related fields in the future.

## Data Availability

Because we have follow-up research and the confidentiality of the research is involved, the data set cannot be made public, but it can be obtained by contacting the author for reasonable reasons.

## Footnotes

1 https://www.gov.cn/xinwen/2022-12/07/content_5730475.htm

2 https://m.gmw.cn/baijia/2022-12/14/1303223656.html

3 https://www.gov.cn/xinwen/2022-11/11/content_5726122.html The Chinese central government promulgated the “20 Policies for Epidemic Prevention” and hopes to scientifically manage the epidemic containment policy through the new policy. However, local governments are worried that the development of the epidemic will affect their political performance, which has led to the implementation of the “20 epidemic prevention policies” by local governments (Random closure of schools and classes, suspension of production, unauthorized blocking of traffic, random “silent” management, random sealing control, long time not unsealing, random suspension of medical treatment, etc.). Residents are dissatisfied with the actions of local governments.

4 “Spring Festival” is the most important festival of the year in China

5 National Health Commission, a idiom used by Chinese netizens.

6 This information comes from the official news conference of WHO, which was broadcasted live by the Chinese government to announce this information to the public. Visit www.who.int/ for more details.
